# Cytokines and inflammatory mediators: Markers involved in interstitial damage to the pancreas in two dengue fever cases associated with acute pancreatitis

**DOI:** 10.1371/journal.pone.0262785

**Published:** 2022-01-18

**Authors:** Felipe de Andrade Vieira Alves, Lucca de Lima S. Oliveira, Natália Gedeão Salomão, David William Provance, Carlos Alberto Basilio-de-Oliveira, Rodrigo Basílio-de-Oliveira, Leandro Junqueira Moragas, Jorge José de Carvalho, Ronaldo Mohana-Borges, Kíssila Rabelo, Marciano Viana Paes

**Affiliations:** 1 Laboratório Interdisciplinar de Pesquisas Médicas, Instituto Oswaldo Cruz, Fundação Oswaldo Cruz, Rio de Janeiro, Brasil; 2 Centro de Desenvolvimento Tecnológico em Saúde, Fiocruz, Rio de Janeiro, Brasil; 3 Anatomia Patológica, Universidade Federal do Estado do Rio de Janeiro, Rio de Janeiro, Brasil; 4 Laboratório de Ultraestrutura e Biologia Tecidual, Universidade do Estado do Rio de Janeiro, Rio de Janeiro, Brasil; 5 Laboratório de Genômica Estrutural, Instituto de Biofísica Carlos Chagas Filho, Universidade Federal do Rio de Janeiro, Rio de Janeiro, Brasil; Chang Gung University, TAIWAN

## Abstract

Dengue viral (DENV) infections can lead to acute pancreatitis and associated tissue damage. This study examined the pancreas from two fatal cases of DENV for histopathological changes as well as for the detection of cytokines, and other inflammatory mediators. Tissue sections were prepared for examination by ultrastructural and histopathological techniques. Sections from the pancreas of non-infected individuals were prepared in parallel as a control. The presence of viral replication in macrophages was detected by co-staining for the proteins NS3 and CD68 by immunofluorescence. Immunohistochemistry was used to detect cells that expressed cytokines and inflammatory mediators to characterize the inflammatory response. Edema, acinar necrosis and fibrosis areas associated with a mononuclear infiltrate were found in infected tissues. The major site of virus replication appeared to be macrophages based on their exclusive presentation of the viral protein NS3. Pancreatic tissues from the infected individuals also displayed increased levels of high mobility group box-1, caspase-3, gelatinase B and tumor necrosis factor alpha compared to controls. The presence of virus replicating macrophages in the pancreas was associated with multiple changes in tissue structure that included elevated levels of cytokines and inflammatory markers that may differentiate acute pancreatitis due to DENV infections from other causes.

## Introduction

Dengue is an arbovirus disease that affects people in more than a hundred countries worldwide [1]. The etiologic agent, dengue virus (DENV), comprises of four antigenically distinct serotypes transmitted mainly by the bite of the *Aedes aegypti* and *Aedes albopictus* mosquitoes [2]. From an epidemiological perspective, it is estimated that 2.5 billion people live in endemic areas with approximately 50 million infected annually that contribute in 25,000 deaths per year [3].

Infection of individuals with any one of the DENV serotypes can cause a wide variety of symptoms from asymptomatic to low grade dengue fever to more serious cases of severe dengue that most often culminate with hospitalization and even death [4]. Some of the complications associated with severe dengue include encephalitis, myocarditis and pancreatic involvement [5]. It is known that other viral infections can cause acute pancreatitis (AP), such as hepatitis B and A, chickenpox, mumps, and this is a complication that only occurs in rare cases of severe dengue [6,7].

The correlation between severe dengue and the development of acute pancreatitis have been demonstrated [8,9]. The largest related series between pancreatitis and dengue infection was during an outbreak that occurred in 2002 in Taiwan, in which three patients were diagnosed with dengue hemorrhagic fever and acute pancreatitis [10]. Over the years some theories have been raised to describe the mechanisms evolved in the development of AP in viral infections, such as pancreatic acinar cells apoptosis and necrosis, enzyme activation, self-digestion of the pancreatic tissue and others. However, the etiopathogenesis of acute pancreatitis in viral infections is still unclear [7,11].

In a previous report by our group, DENV antigens and cytokines such as tumor necrosis factor alpha (TNF-α), transforming growth factor beta (TGF-β) and interleukin 10 (IL-10), were detected in organs such as liver, lungs and kidneys [12]. This cytokine profile was also observed in studies on acute pancreatitis [13–15]. Due this commonality, we considered the potential that both viral proteins and these cytokines could be present in the pancreas of individuals infected with DENV. Furthermore, we assessed fibrosis and tissue remodeling by the expression of TGF-β and MMP-9 collagenase [16]. Finally, caspase-3, a key mediator in mitochondrial events of apoptosis [17], and high mobility group box-1 (HMGB-1), a protein that have an important role in transcription and activation in pro-inflammatory response, also correlated with the severity of pancreatitis [18,19], were investigated. In this context, the research of associated damages and cytokines described above, alongside with mediators, can provide new insights about the immunopathogenic mechanisms triggered during viral induced acute pancreatitis.

## Materials and methods

### Ethical procedures

All procedures associated with this study were approved by the Ethics Committee of the Oswaldo Cruz Foundation/FIOCRUZ (CAEE: 47525115.3.0000.5248).

### Clinical history of patients infected with DENV

During the 2002 Brazilian outbreak of DENV infections in Rio de Janeiro, our group obtained samples of pancreatic tissue from two patients after their death in São Vicente de Paula and Clementino Fraga Filho/UFRJ Hospitals. Each had been admitted with Dengue fever symptoms and were confirmed with anti-DENV IgM antibodies. As negative controls, samples were obtained in the same time period from the pancreas of two fatalities presenting no signs of a dengue or other infection, or any pancreatic disease. More details regarding cases one and two are described in a previous report [20].

#### Case 1

A 63-year-old diabetic male self-medicating with acetyl salicylic acid (100 mg) and Daonil, suddenly presented with headache, myalgia and abdominal pain. Admitted to Hospital São Vicente de Paulo, the physical examination revealed blood pressure of 140/80 mmHg and skin rash. On the fourth day of hospitalization, the patient presented diarrhea, thrombocytopenia (platelets 79,000 / mm^3^), leukocytopenia, hemoconcentration (hematocrit: 59%), and elevated serum levels of the enzymes amylase 940 IU/dL and lipase 192 IU/L. Ultrasonography revealed peri-hepatic and peri-pancreatic collections confirmed by computed tomography of the abdomen. There was also an enlarged heart, slight opacities in the left lung with marginal pleural reaction and distension of the gallbladder. The individual presented a progressive worsening of the clinical condition, progressing to shock with severe pulmonary congestion followed by death with a clinical diagnosis of hemorrhagic dengue, ischemic cardiomyopathy and pancreatitis.

#### Case 2

Female patient, 21 years old, obese, presented fever, myalgia and headache for 8 days with symptoms progressing to metrorrhagia, nausea, abdominal pain, vomiting and diarrhea. Prior to hospitalization, the patient was examined at another health service with a hypothetical diagnosis of dengue due to severe leukopenia and thrombocytopenia (platelets 10,000 / mm^3^). Later, she was admitted to the intensive care unit (ICU) of Hospital Universitário Clementino Fraga Filho with respiratory failure with elevated serum levels of glucose 158 mg/dL; aspartate aminotransferase 149 IU/L and alanine aminotransferase 66 IU/L. Ultrasonography revealed and pancreatic liquid collections. The clinical picture evolved to multiple organ failure and refractory shock, culminating in her death.

### Histopathological analysis

Pancreatic tissue samples from necropsies were fixed and treated as described in a previous work [21,22]. Briefly, Tissue sections (4 mm thick) were mounted onto glass sides, deparaffinized in three baths of xylene and rehydrated with decreasing concentrations of ethanol (100 to 70%) before staining. Sections were stained with Hematoxylin and Eosin or Picro Sirius Red and then slides were prepared for visualization under a Nikon ECLIPSE E600 microscope and digital images captured using Image-Pro Plus software version 7.

### Electron Microscopy procedure

The pancreatic tissue fragment from one fatal case and control were fixed with glutaraldehyde (2.5%) in sodium cacodylate buffer (0.1 M, pH 7.2), post-fixed with 1% buffered osmium tetroxide, dehydrated in an acetone series (30, 50, 70, 90, and 100%) and embedded in EPON that was polymerized at 60°C for 3 days. Ultrathin sections (50–70 nm) contrasted with uranyl acetate and lead citrate and visualized using a JEOL 1001 transmission electron microscope (Jeol Ltd.).

### Immunohistochemistry

For the detection of cytokines, inflammatory mediators and cell populations by immunohistochemistry, sections were treated as described before [21]. The slides were incubated overnight at 4°C with the primary antibodies that recognized HMGB1 (Abcam, UK; dilution 1:200), Caspase-3 (Abcam, UK; dilution 1:100), TGF-β (Abbiotec, CA, USA; dilution 1:300), MMP-9 (Santa Cruz Biotechnology, CA, USA; dilution 1:200), TNF-α (Santa Cruz Biotechnology, CA, USA; dilution 1:200) or IL-10 (Abbiotec, CA, USA; dilution 1:100) and prepared for visualization under a Nikon ECLIPSE E600 microscope.

### Immunofluorescence assay

The pancreatic tissue fragment from the fatal case one and control were fixed and treated as described earlier [21]. Co-staining was performed overnight at 4°C with a mouse monoclonal anti-NS3 (Expressed in *Escherichia coli*, purified and inoculated in BALB / c mice; dilution 1:200) and a rabbit monoclonal anti-CD68 (Santa Cruz Biotechnology, CA, USA) at the same dilution. Sections were followed by a sequential incubation with Alexa 555-conjugated goat anti-rabbit IgG followed by Alexa 488-conjugated rabbit anti-mouse IgG (Sigma). Slides were analyzed using a Zeiss LSM 510 Meta confocal microscope (Carl Zeiss).

### Morphometry, quantification and statistical analysis

Fields on the slides were imaged using a Cool SNAP-Procf Color camera coupled to a Nikon ECLIPSE E600 microscope. For the semiquantitative analysis of the degree of inflammatory infiltrate, an arbitrary scale of 0–4 (0 = none; 1 = mild; 2 = moderate; 3 = severe; 4 = very severe) was adopted, where the degrees were implemented according to the infiltrate observed in each quadrant. Collagen morphometry was performed with 20 fields were randomly acquired at 400x magnification from across the samples (infected and controls) and the area of collagen was measured to calculate the percentage of collagen area (collagen area/total area of the image). Quantification of positive cells by Immunohistochemistry were performed for each specific antibody stain, images from 20 random fields were acquired at 1000x magnification using the software Image Pro version 7 from samples originating from all samples. The number of positive cells were quantified in each of the 20 fields and the mean number of positive cells per field was calculated. All image acquisitions were performed by an individual blinded to the diagnosis associated with the tissue sample. Figures present representative fields to best convey the quantification results. Data were analyzed with GraphPad Prism software v 6.0 (GraphPad Software) using non-parametric statistical tests. Significant differences between groups (DENV-patients and controls) were determined using Mann-Whitney test with p < 0.05.

## Results

### Histopathological analysis

**Fig 1:**
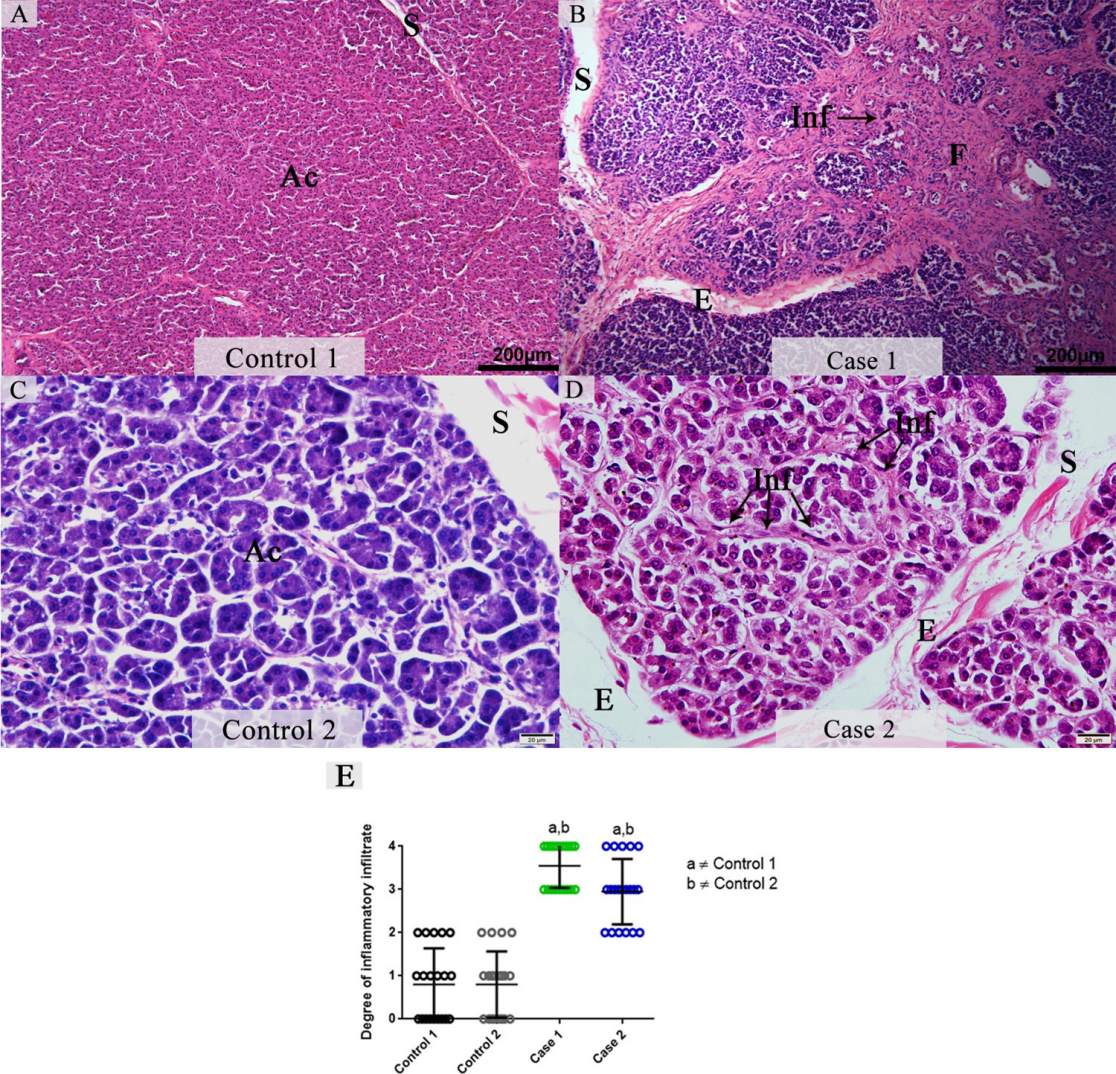
Histopathological aspects of pancreatic tissues from DENV fatal cases in comparison to non-dengue controls. (**A and C**) Representative images of control pancreatic tissues displaying regular parenchyma. (**B and D**) Images from pancreatic tissues obtained from two DENV fatal cases presenting edema and extensive areas of fibrosis associated with mononuclear inflammatory infiltrate. (**E**) Semiquantitative analysis of the degree of inflammatory infiltrate. Data represent the mean ± SDM. Statistically significant differences (p < 0.05) between each fatal dengue case and each control (a and b). Both cases revealed a significant increase in the infiltrate degree compared to controls. (Ac) Pancreatic acini; (S) Interlobular septa; (E) Edema; (Inf) Infiltrate and (F) Fibrosis.

The evaluation of pancreas tissue from cases of fatal dengue infections exhibited alterations such as diffuse edema with an increase in the interlobular septum and extensive areas of fibrosis. In association with the areas of fibrosis, a mononuclear infiltrate was observed mainly in the interstice (Fig 1B and 1D). There was an approximate 3.5-fold increase in pancreatic mononuclear infiltrate in the pancreas of fatal dengue cases compared to non-dengue samples that showed an altered inflammatory profile (Fig 1E). The histopathological analysis indicated an absence of changes in the control tissues parenchyma. As expected, we observed well preserved acini and interlobular septa with regular thickness (Fig 1A and 1C).

### Ultrastructural aspects

**Fig 2:**
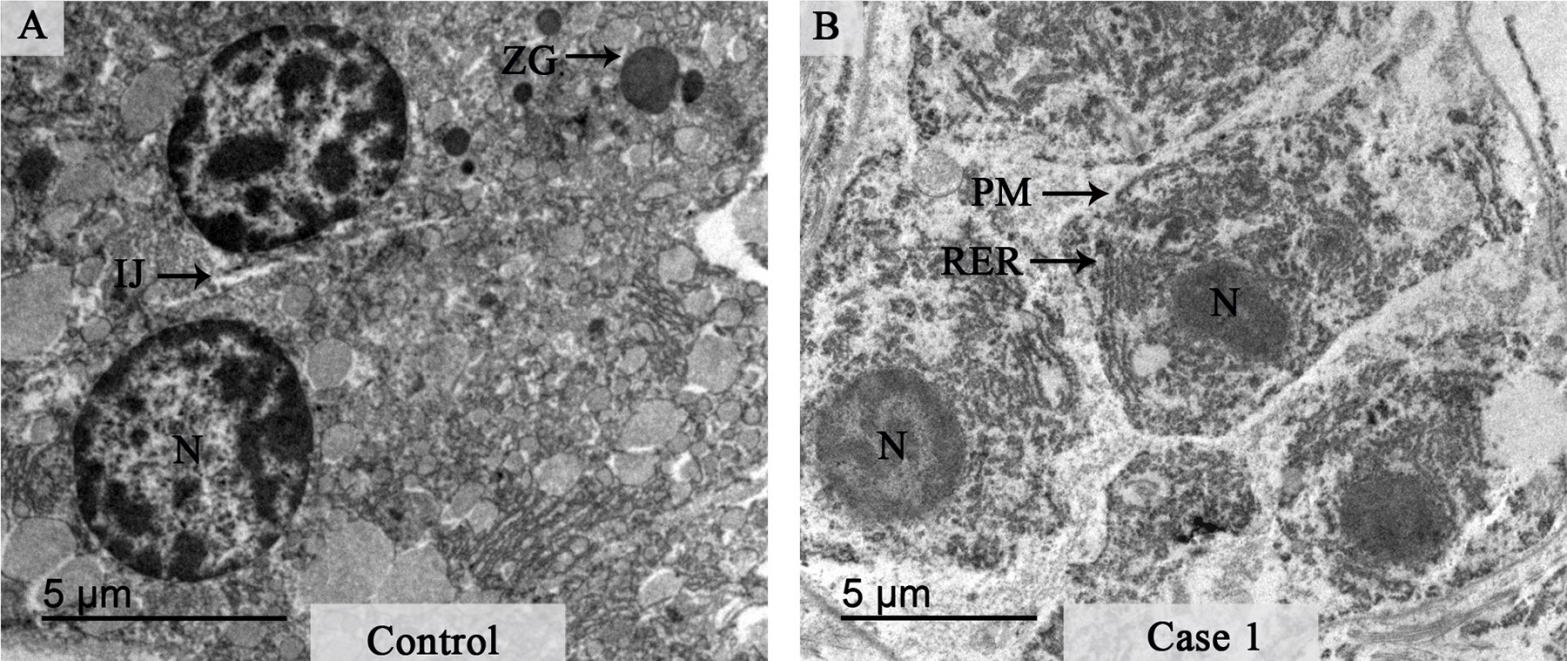
Ultrastructural aspects of pancreatic tissue from control and dengue-infected cases. (**A**) Electron micrograph of pancreatic acini in control tissue with regular structural features. (**B**) Electron micrograph of the infected pancreatic tissue that shows a loss of plasma membrane integrity along with rarefied cytoplasm. Interstitial junction (IJ); Zymogen granules (ZG); Nucleus (N); Rough Endoplasmic Reticulum (RER); Plasma membrane (PM).

The ultrastructural analysis of a control pancreas sample revealed normal aspects in the acinar cells that presented well-preserved zymogen granules, nucleus with condensed chromatin and regular interstitial junction (Fig 2A). In a representative image from a sample from a fatal case tissue (Case 1), the zymogen granules and organelles were absent (Fig 2B). In addition, cells showed rarefied cytoplasm and a rupture of the plasma membrane that suggested cellular necrosis. Cells also presented prominent rough endoplasmic reticulum and pyknotic nucleus.

### Detection of viral antigens and replication in hyperplasic macrophages from interstitial pancreas of dengue-infected patients

**Fig 3:**
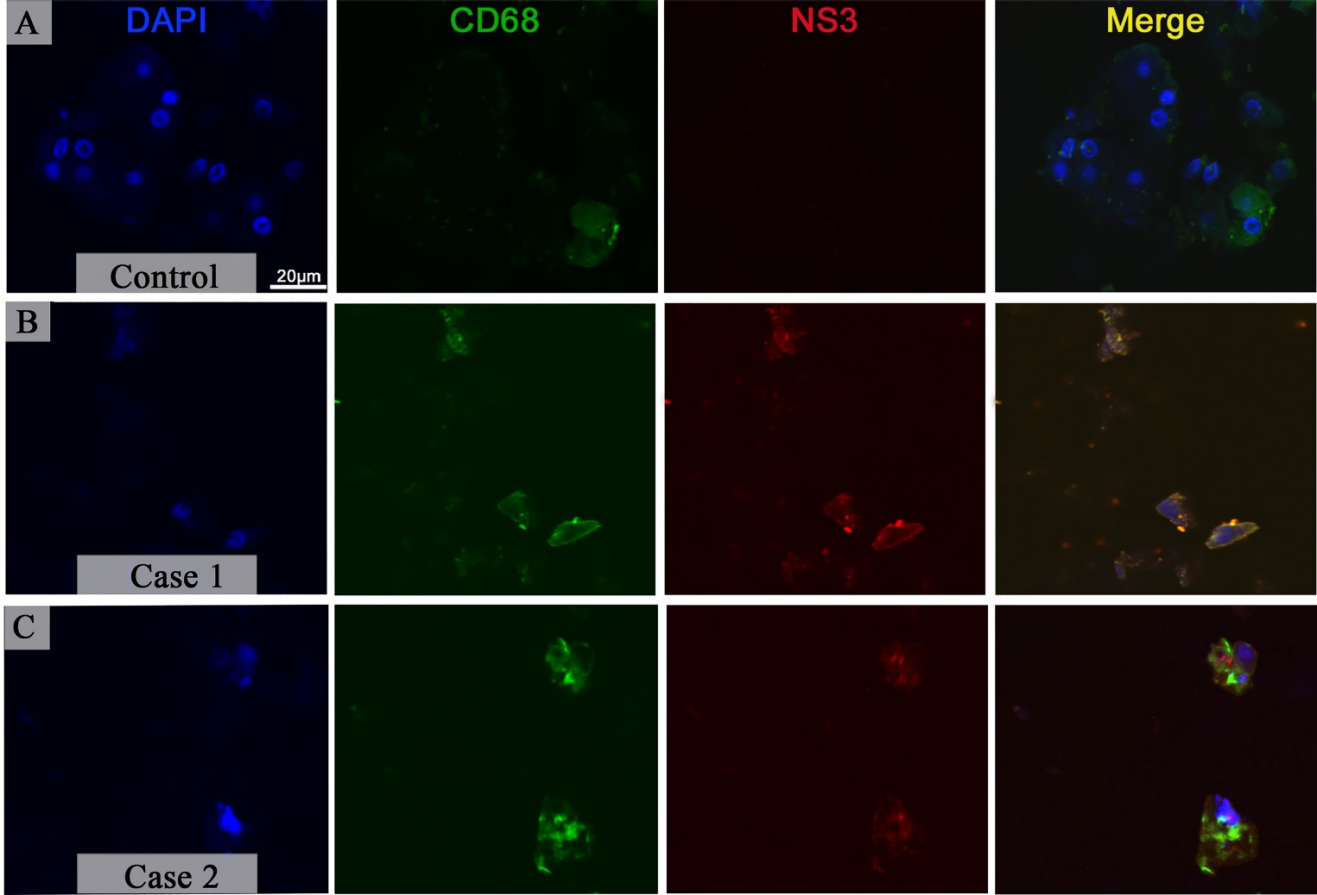
Presence of the viral protein NS3 in macrophages of pancreatic tissue in dengue-infected cases. (**A**) Macrophages were detected in control tissue although the expression of NS3 was not observed. (**B-C**) Tissue from fatal cases of dengue infection presented macrophages with an irregular morphology and the protein NS3 of the DENV in their cytoplasm. Nuclei were stained using DAPI.

To determine if macrophages are sites of viral replication in pancreas, a co-staining between non-structural NS3 protein and CD68^+^ cells was performed. The incubation with DAPI (fluorescent blue) revealed preserved nuclei in control tissues, and positive CD68^+^ cells were present (fluorescent green) although the expression of NS3 was not detected as expected (Fig 3A). In contrast, the expression of the DENV NS3 protein (fluorescent red) was readily observed in tissues from infected patients that was mainly present in the cytoplasm of CD68^+^ macrophages (Fig 3B and 3C).

### Evaluation of the fibrosis by collagen quantification in pancreatic tissues

**Fig 4:**
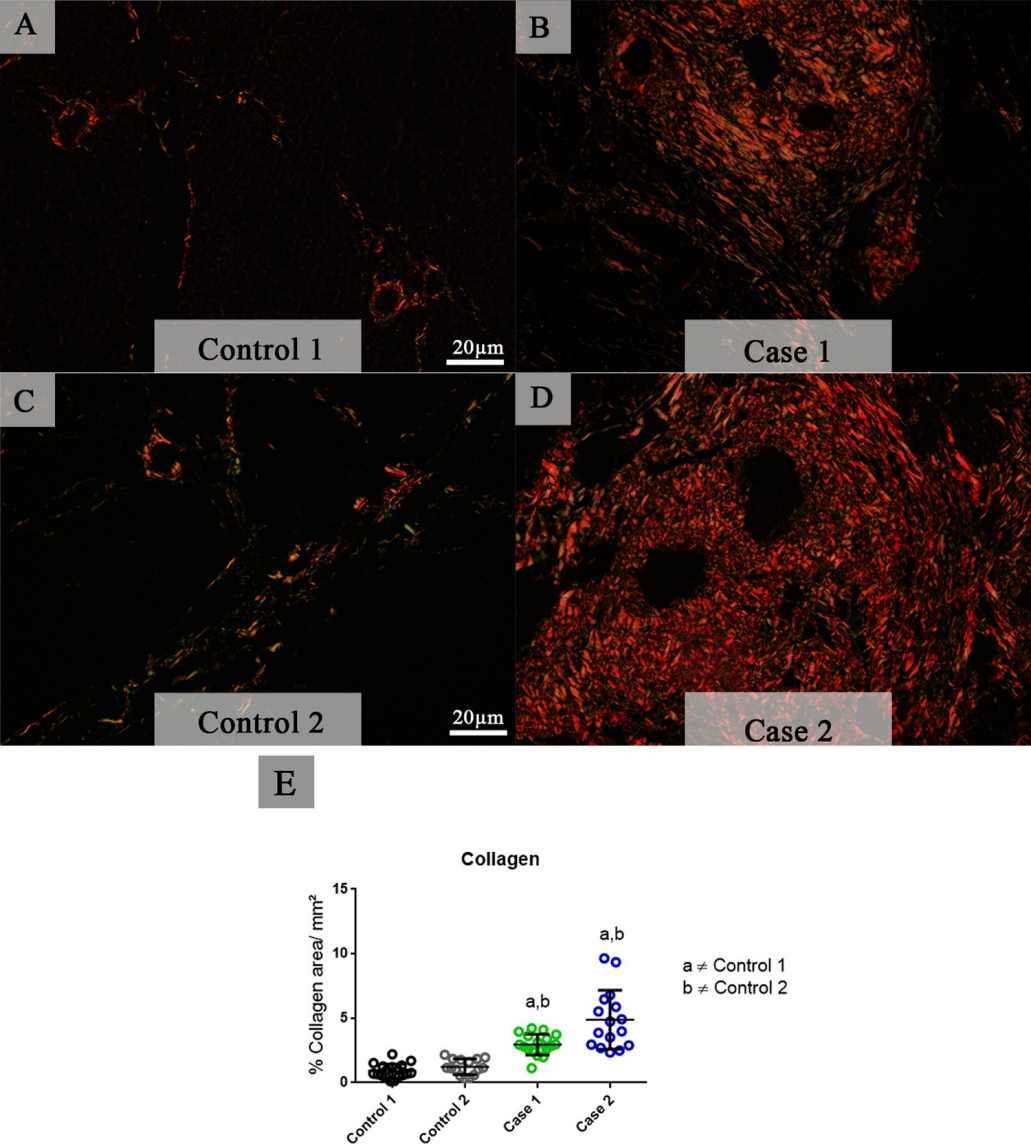
Analysis of tissue fibrosis by collagen expression. (**A and C**) Control tissues revealing a constitutive collagen deposition as well as vessels with a regular thickness and preserved pancreatic parenchyma. (**B and D**) Fatal cases of DENV infection with extensive areas of interacinar fibrosis, vessels with thickened caliber and deposition of abundant collagen around ducts and vessels. (**E**) Both cases presented a significant increase in collagen deposition compared to controls, which revealed damage in the infected pancreatic tissue. Data are represented as mean ± SD. (a and b) indicate differences that are statistically significant between individual specimens (p < 0.05).

To analyze fibrosis in pancreatic tissues, samples were stained by Picro Sirius red to mark collagen deposition. Control tissues exhibited normal collagen deposition that was present around vessels and displayed a regular thickness (Fig 4A and 4C). In both fatal cases, we observed extensive areas of fibrosis, which corroborate with our previous histopathological findings. Exacerbated interacinar deposition of collagen was observed in dengue cases ending up by disrupting the pancreatic parenchyma. Although, it was also observed collagen deposition around vessels, which had a thickened caliber and ducts (Fig 4B and 4D). A quantitative analysis demonstrated a significant increase in the deposition of collagen in both cases of fatal dengue infection compared to controls (Fig 4E).

### Evaluation of tissue remodeling by TGF-β and MMP-9 expression in pancreatic tissues from DENV fatal cases

**Fig 5:**
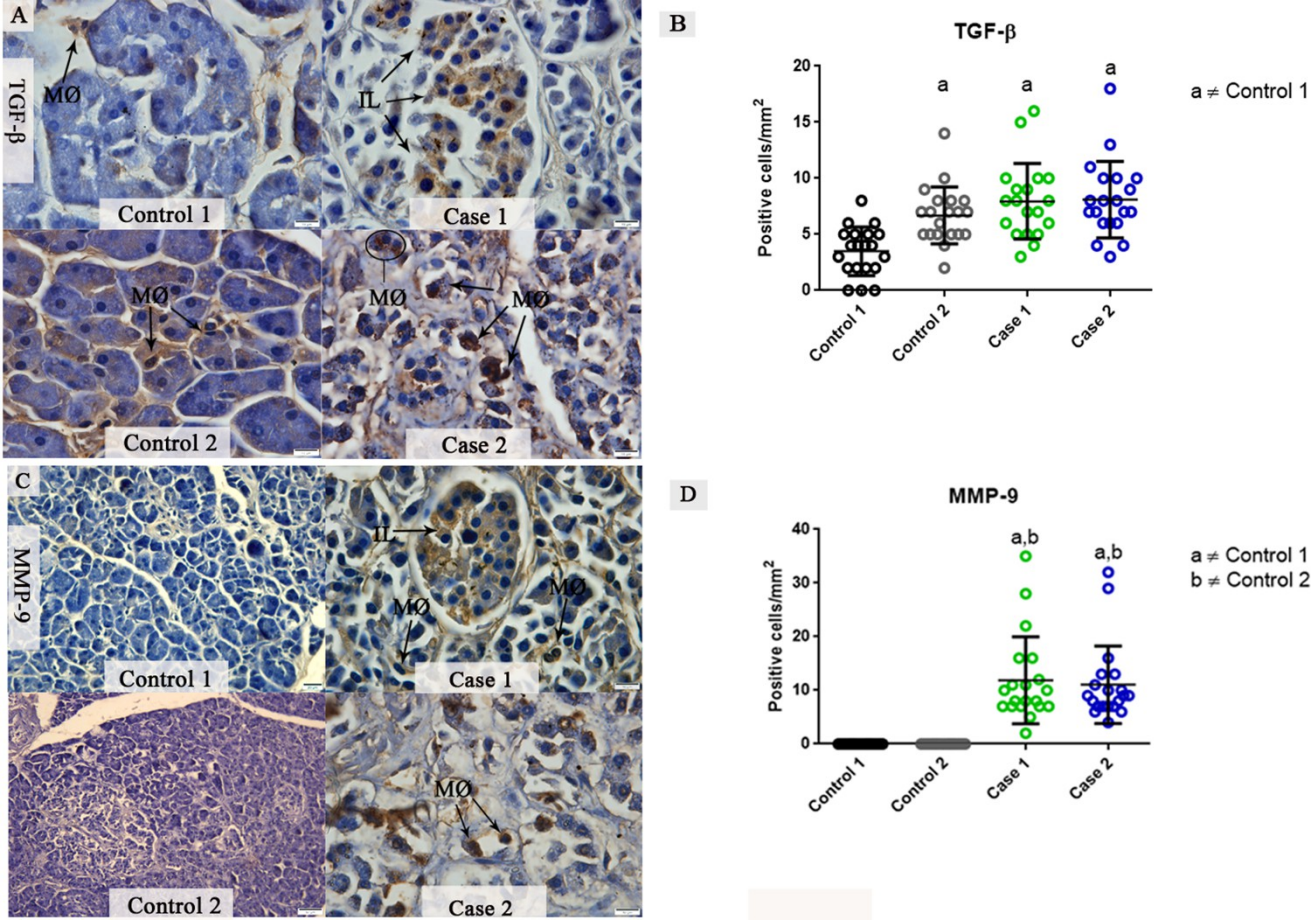
Detection and quantification of the TGF-β and MMP-9 in fatal cases of DENV. (**A**) Detection of TGF-β in pancreatic islet cells and differentiated macrophages in both fatal cases. Control tissue showed a constitutive expression of this cytokine. (**B**) Quantitative analysis of TGF-β expression exhibited a significantly increase in infected tissues compared to control tissues. (**C**) MMP-9 expressed by islet cells and macrophages in DENV fatal cases. The detection of this metalloproteinase was not observed in control tissues. (**D**) The quantitative analysis of MMP-9 expression showed increased levels in both cases. Macrophages (MØ); Pancreatic Islets (IL). Data are represented as mean ± SDM. (a and b) indicate differences that are statistically significant between individual specimens (p < 0.05).

In infected pancreatic tissues, TGF-β expression was evaluated in both activated macrophages and pancreatic islet cells. The presence of TGF-β in the control tissue was also detected, although this cytokine is constitutively expressed at baseline levels in immune cells present in the tissue (Fig 5A). The quantitative analysis of TGF-β levels showed close to a 3-fold increase in this cytokine in tissues from fatal cases of DENV infections compared to the first control tissue. However, the second control sample showed TGF-β levels similar to the infected cases (Fig 5B). MMP-9 was also detected in differentiated macrophages of the pancreatic tissues from both fatal cases. Islet cells expressing MMP-9 were observed in these tissues. No MMP-9 was observed in control tissues (Fig 5C). The quantitative analysis of MMP-9 expression was 2.9 times greater than that detected in the controls, which showed very low signs of MMP-9 (Fig 5D).

### Detection of IL-10 and TNF-α cytokines in pancreatic tissues from DENV fatal cases

**Fig 6:**
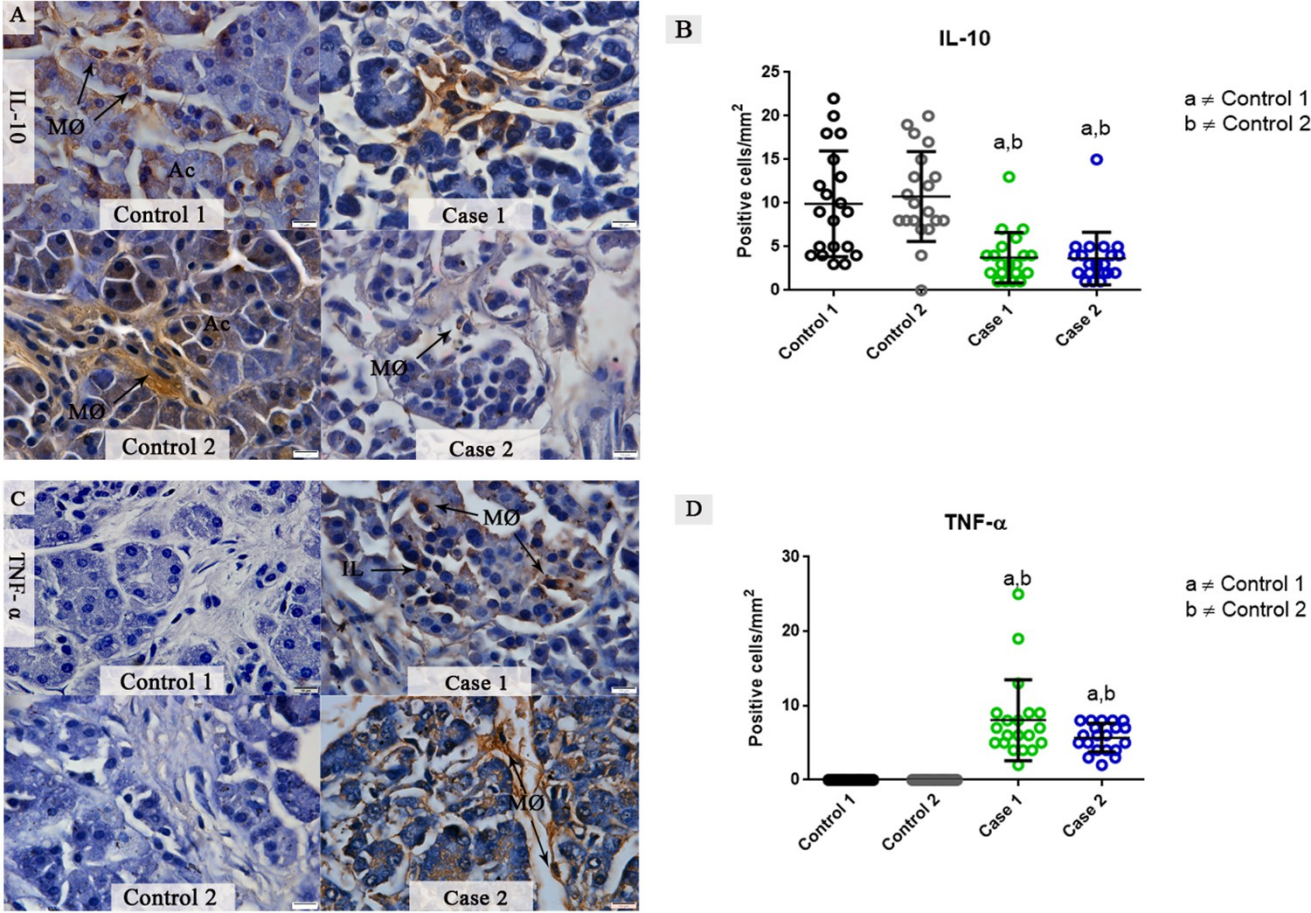
Detection and quantification of the cytokines TNF-α and IL-10 in fatal cases of DENV. (**A**) Detection of IL-10 in focal areas and few macrophages in both fatal cases. Control tissues exhibited a high expression of IL-10. (**B**) Quantitative analysis of IL-10 showed a significantly decrease in fatal cases compared to controls. (**C**) Detection of TNF-α in macrophages of pancreatic islets and underlying connective tissue. TNF-α was not detected in control tissues. (**D**) Quantitative analysis of TNF-α revealing an increase in the expression of this cytokine compared to controls. Data are represented as mean ± SDM. (a and b) indicate differences that are statistically significant between individual specimens (p < 0.05).

In pancreatic control tissues, the expression of the anti-inflammatory cytokine IL-10 was diffuse in pancreatic tissues that was limited to acinar cells and resident macrophages. IL-10 was also detected, but just in some focal areas of the infected pancreatic tissues (Fig 6A). TNF-α was not detected in control tissues, but in infected pancreas, it was mostly seen in infiltrated macrophages in pancreatic islets and the underlying connective tissue (Fig 6C). A quantitative analysis of IL-10 expression exhibited a 2-fold increase in control 1 when compared to fatal cases. Control 2 exhibited a 2.2-fold increase in this cytokine when compared to fatal cases (Fig 6B). TNF-α had an average of 8.05 positive cells / mm^2^ expressed in fatal case 1, while case 2 exhibited an average of 5.70 positive cells / mm^2^ compared to controls, where this cytokine was not observed (Fig 6D).

### Investigation of apoptotic mediators present in pancreas tissues of dengue fatal cases

**Fig 7:**
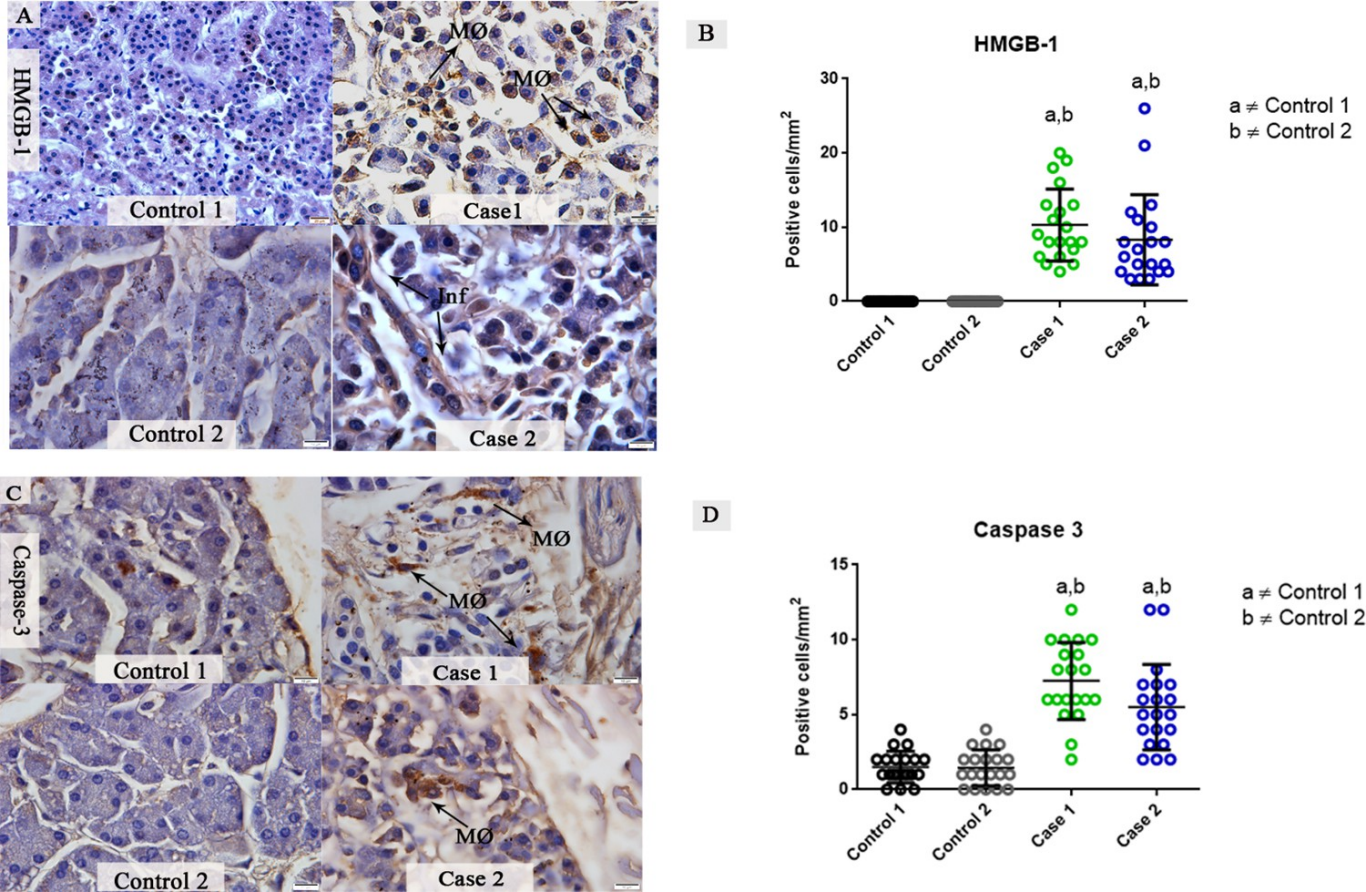
Detection and quantification of the HMGB-1 and caspase-3 proteins in fatal cases of DENV. (**A**) A constitutive expression of HMGB-1 observed in pancreatic control tissue, while pancreatic infected tissues exhibited a diffuse expression of HMGB-1, mainly in the mononuclear infiltrate. (**B**) Quantitative analysis of HMGB-1 expression, revealing a significant increase compared to control tissues. (**C**) Detection of caspase-3 in macrophages in both DENV fatal cases. Control tissues exhibited a constitutive expression of caspase-3. (**D**) Quantitative analysis of caspase-3 expression, revealing a significant increase in both cases compared to controls. Macrophages (MØ); Infiltrate (Inf). Data are represented as mean ± SDM. (a and b) indicate differences that are statistically significant between individual specimens (p < 0.05).

The expression of cytoplasmatic HMGB-1 was observed in the interstitial infiltrate of infected pancreatic tissues, mainly in macrophages. On the other hand, control tissues exhibited a constitutive expression of HMGB-1, however, HMGB-1 expression by immune cells was not observed (Fig 7A). Quantitative analysis of cells expressing HMGB-1 showed an average of 10.30 positive cells / mm^2^ in fatal case 1, while fatal case 2 revealed an average of 8.3 positive cells / mm^2^ (Fig 7B). The protein caspase-3 was also evaluated as an apoptosis marker. In both fatal cases tissues, activated macrophages showed expression of caspase-3. In control tissues, a constitutive expression of caspase-3 was observed, which can reflect a physiological apoptosis process (Fig 7C). The quantitative analysis of caspase-3 reveals an almost 3-fold increase in the fatal case 1 compared to control tissues (Fig 7D), and fatal case 2 showed a 2.4-fold increase compared to controls.

## Discussion

Acute pancreatitis is caused by an inflammatory response that can be triggered by several agents including viruses, although the etiopathogenesis of this disorder in such circumstances remains unclear [7], especially in dengue cases. In the present work, we examined pancreatic tissues from two dengue fatal cases concerning their histopathological and ultrastructural aspects that included viral protein detection as well as an analysis of their inflammatory state and cell death. According to the 2013 American College of Gastroenterology (ACG) guidelines, the diagnosis of acute pancreatitis requires the presence of at least two of the following characteristics: 1) typical abdominal pain, 2) amylase and/or lipase greater than three times normal, and 3) characteristic findings on imaging exams (ultrasonography, abdominal computed tomography (CAT), magnetic resonance imaging) [23,24]. The increased expression of amylase and lipase enzymes in the patient’s serum of case 1, associated with the peri-hepatic and peri-pancreatic collections observed in the ultrasonography confirms the diagnosis of acute pancreatitis [25]. Although the serum levels of pancreatic enzymes in case 2 are unknow, the ultrasonography presenting liquid collection and abdominal pain characterized the acute pancreatitis. The histopathological features such as diffuse edema, mononuclear infiltrate and acinar necrosis are similar to findings from previous studies on acute pancreatitis [26–28]. We observed an increase in the areas of diffuse edema within the interlobular septum that was associated with a mononuclear infiltrate and extensive areas of fibrosis. These observations were expected since severe dengue infections are associated with an increase in vascular permeability leading to plasma leakage and consequently edema, which is a common finding in acute pancreatitis. Also, the direct cytopathic effect to acinar cells is proposed to be a mechanism of injury and development of acute pancreatitis [29–31]. The infiltrate observed in the interstice was predominantly composed of macrophages, cells that are described as playing a major role in dengue pathogenesis and in acute pancreatitis. It has been proposed that these cells contribute an important role in the cytokine storm and can be related to the process of vascular leakage though the secretion of cytokines such as TNF-α and HMGB-1 [32–34], which was also noted in our work.

The fibrosis process of the pancreas is commonly seen in chronic pancreatitis. However, a study with cerulein-induced acute pancreatitis in mice reveals that the induction of acute pancreatitis repeatedly can promote the development of fibrosis by the excessive TGF-β activity, a cytokine that can be a link between acute and chronic pancreatitis [35]. Thus, diabetes and obesity as comorbidities can contribute to the pancreatic fibrosis due to the intense inflammatory environment and secretion of TGF-β by macrophages and other cells. The Picro Sirius Red, a special stain utilized to identify collagens I and III revealed an intense deposition of collagen in both cases, that may be intensified to these comorbidities [36–39]. Although in the cases evaluated the levels of MMP9 were high, this enzyme only degrades collagen IV, which is not evidenced in the picrosirius staining [40].

The absent or reduced numbers of zymogen granules found in the pancreatic tissues suggest that the synthesis of secreted proteins is impaired [41]. Alterations such as dilatation of the RER, loss of organelles and rupture of plasmatic membrane indicates necrosis of the acinar cells and were in agreement with findings observed in other work [42].

Previous investigations of our group detected the NS3 protein in several organs of the patients studied in this work, such as kidneys, liver, lungs, spleen, and especially in Kupffer cells and macrophages [20]. The detection of this nonstructural protein can identify sites of viral replication in a tissue since this proteins is not secreted [43]. Here, the immunofluorescence revealed the presence of the protein NS3 in the pancreatic tissue, inside macrophages, which strongly indicate that viral replication occurs within these cells. These findings are in accordance with other studies and supports the hypothesis that macrophages play an important role in DHF pathogenesis [44,45].

As observed in the histological analysis, the pancreatic tissues presented a strong process of fibrosis. Transforming growth factor beta and metalloproteinases are known to be key modulators of the extracellular matrix production and can be involved in the process of fibrosis and tissue remodeling. An important role for TGF-β has been suggested since this cytokine inhibits the production of MMPs that degrade collagen and, consequently, enhances the formation of fibrous tissue [16]. Furthermore, the inhibition of TGF-β decreases pancreatic fibrosis and prevent acinar cell death [46]. In spite of high levels of TGF-β following increase of collagen being correlated with a variety number of diseases, including acute pancreatitis, it is also a cytokine expressed in normal pancreatic tissue, playing an important role in pancreas organogenesis, even in the mature pancreas [47–50]. Based on the quantitative analysis from our study, we postulate that with the intense inflammatory process, the levels of TGF-β were not sufficient to avoid the tissue repair process, resulting in fibrosis in both DENV fatal cases. Consistent with this hypothesis was the observed increased levels of MMP-9 that reveals that the levels of TGF-β did not interfere with the production of this enzyme, which also have been produced by the excess of immune cells infiltrating the tissue. A murine model of acute coxsackievirus B4-induced pancreatitis have also shown increased levels of MMP-9 [28]. In addition, several studies have shown that the levels of MMP-9 can be correlated with the severity of the disease, although the exact function of MMP-9 in acute viral pancreatitis remains elusive [51,52].

TNF- α and IL-10 are cytokines that have been well studied in acute pancreatitis [13]. The release of TNF- α by macrophages, its main source, might play a crucial role in the pancreas inflammation by inducing the recruitment of other macrophages. These can produce then many of the mediators involved in vascular permeability that can aggravate the effect on the tissue and perpetuate the inflammatory response. TNF- α, alongside with MMP-9, correspond to important mediators that drives to the disruption of endothelial barrier membranes, causing an increase in permeability and consequently edema. Several researches have been linking the upregulation of MMP-9 by TNF—α expression, and the levels of these two mediators have been correlated with the severity of acute pancreatitis as described earlier. The mechanisms behind the upregulation of MMP-9 are uncertain, although, some theories have been proposed in the past years. One hypothesizes that a complex between TNF- α/fibronectin and laminin, major adhesive glycoproteins of extracellular matrix, could stimulate the adhesion of monocytes and MMP-9 secretion through TNF receptor–binding [53–56]. It has already been shown that MMP-9 is overexpressed by monocytic cells in granulomas from tuberculosis cases and the TNF- α blockade decreased the expression of MMP-9 by 50% revealing that TNF- α is a key cytokine in monocyte-derived MMP-9 secretion [57]. Studies in rats have demonstrated that acinar cells can respond to TNF- α and could mediate apoptosis, which would contribute to the development of pancreatitis and necrosis of the pancreatic tissue. In experimental studies, the cytokine IL-10 negatively modulates the secretion of pro-inflammatory cytokines and the low levels of this cytokine found in the pancreatic tissues of both fatal cases could have contributed to a pro-inflammatory environment [58–60].

HMGB-1, a protein that can trigger inflammation by its released from necrotic and immune cells, was increased in the pancreatic tissue of fatal DENV cases. In a previous report, dengue cases showed increased levels of HMGB-1 in several tissues that revealed the important role of this protein in dengue pathogenesis [61]. It is known that HMGB-1 can activate the NF-kB pathway, which acts in the induction of the expression of many cytokines that amplify a pro-inflammatory response. The elevated levels of HMGB-1 could explain the previously observed increased levels of NF-kB observed in DENV infections [14,62]. A study on cerebral ischemia in rats also postulates that HMGB-1 can upregulate the expression of MMP-9 although this mechanism has not been determined in the pancreas [63]. These observations alongside with ultrastructural findings and the increased detection of capase-3, a key protein in mitochondrial events of apoptosis, suggests a strong inflammatory response resulting in necrosis/apoptosis of these tissues [64,65].

In this research, the data suggest that there was an ongoing inflammatory response in the pancreas of DENV infected tissues. This characterization was possible due to multiple lines of evidence that included the observation of a mononuclear infiltrate, higher counts of macrophages expressing TNF-α and HMGB-1 and histopathological and ultrastructural changes. Additionally, low levels of anti-inflammatory cytokines (IL-10 and TGF-β) revealed an apparent strong pro-inflammatory response and may suggest another fibrogenic pathway for the abundant fibrous tissue observed.

## Conclusions

In conclusion, the present study has provided evidence for an intense inflammatory response in pancreatic tissues of fatal DENV infections and can be an aggravating factor for the development of pancreatitis. Furthermore, local inflammation leads to organ tissue remodeling, with damage to the structure which can lead to functional damage to patients and clinicians. As the cases analyzed are rare, further studies are needed to understand pancreatic changes in fatal cases of dengue.
